# Supplementary material for: Patterns of Intron Gain and Loss in Fungi
Source: PLoS Biol. 2004 Nov 30;2(12):e422. doi: 10.1371/journal.pbio.0020422 (PMC532390; doi:10.1371/journal.pbio.0020422)
Supplement: Table S1 — Also available at http://genes.mit.edu/NielsenEtAl/. (4.3 MB ZIP). [file pbio.0020422.st001.zip › NielsenEtAl/html/810.html]

AN4317.1.NCU04063.1.MG00641.1.FG09271.1


```
 CLUSTAL W (1.82) Multiple Sequence Alignments - Introns Inserted


Sequence 1: NCU04063.1	288 aa
Sequence 2: FG09271.1	428 aa
Sequence 3: MG00641.1	289 aa
Sequence 4: AN4317.1	309 aa
Alignment Length: 436 aa
Number Identitical Residues: 178 aa
Alignment Score (without introns) 8758


MG00641.1 	--------------------------------------------~---------------
NCU04063.1	--------------------------------------------~---------------
FG09271.1 	MALRTGAICESVLVMTDIPGLMIDESLEGSTSATRSVRHDCVYI1CLGLGGVFVVAARTE
AN4317.1  	--------------------------------------------~---------------
          	                                                            

MG00641.1 	---~------------------------------------------~-------------
NCU04063.1	---~------------------------------------------~-------------
FG09271.1 	RQE2SVFPQIPKVGSRWLVIAPSSYITATVASGEACDRGNWNMNVC2HPFSICFFLRCNS
AN4317.1  	---~------------------------------------------~-------------
          	                                                            

MG00641.1 	------~-~------------MI0HDAVLDYYGRRLATCSSDRTIKIFEVEGETHRLTET
NCU04063.1	------~-~-------------M0HDAVLDYYGRRLATCSSDRTIKIFEIEGESQRLVET
FG09271.1 	GVKSNK~M0SAQVISNSGHEDMI0HDAVLDYYGRKLATCSGDKTIKIFEIEGETQRLVET
AN4317.1  	-----M0A~AAQVISNSGHDDMI0HDAGLDYYGRRLATCSSDKTIKIFEIEGDTHKLVET
          	         ::.  :.:. .. : *** ******:*****.*:******:**::::*.**

MG00641.1 	LKG2HEGAVWCVAW~AHPKYGNILASSGYDGKVFIWREQGG---------AWQKIFDFAL
NCU04063.1	LKG2HDGAVWSVAW~AHPKYGNILASAGYDGKVLIWREQAG---------SWQRIFDFAL
FG09271.1 	LKG2HEGAVWCVAW~AHPKYGNILASAGYDGKVFIWKEQGGQ------SNAWQRIYDFPL
AN4317.1  	LKG2HEGPVWCVEW0AHPKFGTILASSSYDGKVLIWREQHQSSTAPIGSGAWTKVFDFSL
          	*** *:*.**.* * ****:*.****:.*****:**:**  .:::. .:.:* :::**.*

MG00641.1 	HKASVNIVSWSPHESGCLLACASSDGHVSVLEFKDNSFDHQTFLAHGQGVNSVSWAPSTA
NCU04063.1	HKASVNIVSWSPHEAGCLLACASSDGNVSVLEFKDNSWEHNIFHAHGLGVNSVSWAPATT
FG09271.1 	HKASVNIVSWSPHEAGCLLACASSDGNVSVLEFKDNSIDHTTFAAHGLGVNSVSWAPATT
AN4317.1  	HTASVNMISWAPHETGCLLACASSDGHVSVLEFRDNSWTHQIFHAHGMGVNSISWAPAAS
          	*.****::**:***:***********:******:***  *  * *** ****:****:::

MG00641.1 	PGSIISTNATP--AAQRRFVTGGSDNTLKIWSWD~AASAQYRCEEGGVLSGHTDWVLDVD
NCU04063.1	PGSIVSSNPGPGSTGNRRFVTGGSDNLLKIWTFD~PATNGYKLERE-PLAGHTDWVRDVA
FG09271.1 	PGSIVSSAPGPGATGNRRFVTGGSDNVLKIWAFD~PASQTYKQERE-PLTGHTDWVRDVA
AN4317.1  	PGSLVSSNPGIG--QQRRFVTGGSDNLLKIWDYN2PETKTYNATQT--LEGHSDWVRDVA
          	***::*: .  .   :********** **** :: . :  *.  .   * **:*** ** 

MG00641.1 	WSPTVLQKSYIASASQDRTVRIWTSDSSNPGVWQSRVLKEFDTTVWRVSWSLSGNVLAVS
NCU04063.1	WSPTVLQKSYIASASQDKTVRIWTSDAANPGEWKCKVLN-FDAAVWRVSWSLSGNVLAAS
FG09271.1 	WSPTVLQKSYIASASQDKTVRIWTSDPSNPTQWESKVLP-FEAAVWRVSWSLSGNVLAVS
AN4317.1  	WSPSILSKSYIASASQDKTVRVWTADASNPGQWTSQVLE-FDNVLWRVSWSPSGNILAVS
          	***::*.**********:***:**:*.:**  * .:**  *: .:****** ***:**.*

MG00641.1 	SGDNKVTLWKENLKGEWACVNSLED
NCU04063.1	SDNNKVTLWKENLKGEWENVKTIEE
FG09271.1 	GQDNKVSLWKENLRGEWECVKTIEE
AN4317.1  	GGDNKVSLWKENLRGQWEKVKDIEE
          	. :***:******:*:*  *: :*:
```
